# Pharmaceutical Coating and Its Different Approaches, a Review

**DOI:** 10.3390/polym14163318

**Published:** 2022-08-15

**Authors:** Ahmad Salawi

**Affiliations:** Department of Pharmaceutics, College of Pharmacy, Jazan University, Jazan 45142, Saudi Arabia; asalawi@jazanu.edu.sa

**Keywords:** solid dosage form, uniformity in coating process, tablets, polymers, and film coating

## Abstract

Coating the solid dosage form, such as tablets, is considered common, but it is a critical process that provides different characteristics to tablets. It increases the value of solid dosage form, administered orally, and thus meets diverse clinical requirements. As tablet coating is a process driven by technology, it relies on advancements in coating techniques, equipment used for the coating process, evaluation of coated tablets, and coated material used. Although different techniques were employed for coating purposes, which may be based on the use of solvents or solvent-free, each of the methods used has its advantages and disadvantages, and the techniques need continued modification too. During the process of film coating, several inter-and intra-batch uniformity of coated material on the tablets is considered a critical point that ensures the worth of the final product, particularly for those drugs that contain an active medicament in the coating layer. Meanwhile, computational modeling and experimental evaluation were actively used to predict the impact of the operational parameters on the final product quality and optimize the variables in tablet coating. The efforts produced by computational modeling or experimental evaluation not only save cost in optimizing the coating process but also saves time. This review delivers a brief review on film coating in solid dosage form, which includes tablets, with a focus on the polymers and processes used in the coating. At the end, some pharmaceutical applications were also discussed.

## 1. Introduction

Around 1500 BCE, the first reference to the term pill as a solid dosage form came into existence. The first source of pills in ancient Egypt was recorded to be written on papyruses. The pills were made from bread dough, grease, and honey. Pills were made of simple hand-using ingredients like spices or plant powders. In ancient Greece, medicines were termed katapotia [1]. Roman scholars termed Pills as pilula (little ball). In medieval times, pills were coated using slippery substances obtained from plants. By 1800, gelatin capsules were invented. William Brockedon made a machine that can formulate lozenges and pills with the help of pressure on suitable dies [1]. This device compresses the powder without using adhesive into tablets. Professor Brockedon, 1844 in England, developed the first compressed tablets. These tablets were hard, and no reference was found concerning their disintegration time and solubility. In 1871, Messrs Newbery had purchased Professor Brockedon’s business.

The Brockedon method of tablet compression was used by Philadelphian Jacob Dunton to formulate tablets of different formulations, including quinine [2]. In 1872, two brothers, Mr. Henry Bower and John Wyeth built an advanced machine that was not only more advanced than the previous one but also reduced the cost of producing tablets. In 1878, Dr. Robert R. Fuller from New York, for the first time, suggested the concept of loading these molds with medicated milk sugar. Mr. Fraser, in 1883, started to fabricate molded tablets in a completely new concept that we use today. From the start of the 1940s to the 1990s, synthetic and semisynthetic polymers were used for enteric coating. Dextroamphetamine sulfate was the first manufactured by Kline, Smith, and French as sustained release products using the Spansule method [2,3].

Drug carriers were used to incorporate nutraceutical, cosmeceutical, and pharmaceutical formulations. As per the report published in 2015, it was estimated that $178 million were spent on drug delivery systems, which could be increased to $310 billion by the year 2025.

By definition, the carrier means a system capable of incorporating a specific quantity of medicinal agents to increase their efficiency, selectivity, and bioavailability. The system’s efficiency depends on how much the system could bear the protective barrier. The release of the medicinal agents from the carrier system depends upon the rate and shape of kinetics, the viscosity of the media, and the drug release profile. Lipid-based dosage forms, including microspheres and microcapsules, tend to avoid drug leakage after its administration.

One of the basic advantages of incorporating the drug into the carrier system is to protect the drug during its overall stay within the body, starting from its point of administration until it reaches a specific site of action. Dosage forms were designed according to their usage, specificity, and stimulus-based. Nanosystems were extensively employed to incorporate active constituents, including nanospheres, nanocapsules, niosomes, liposomes, and dendrimers.

With artificial variation in drug release profile, drug release at a specific site could be achieved. The use of the carrier system has successfully bypassed the traditional drug delivery system, which has strong gastrointestinal consequences (gastric irritant drugs). Another reason for using a drug carrier system is to prevent the drugs from biodegradation and increase the bioavailability of specific drugs in the target tissue [4].

By 1992, with the advancements in research, a new carrier system made of mesoporous material gained much attention. The system was further utilized for large pore volume, having increased surface area and a well-organized structure. For the first time in 1998, Zhao and their co-workers synthesized Santa Barbara Amorphous (SBA-15), mesoporous silica with a hexagonal arrangement with high thermal stability, pore size, and surface area. Co-condensation and direct synthesis were the two most commonly used methods for functionalization. Much recently, these mesoporous particles gained more attention as nanoparticles due to their small size ranging from 0.6–1 nm. NSAIDs are muco-irritant and were loaded (as an adsorption phenomenon), especially in SBA-15, due to their nanosized. Once SBA-15 [5] was treated with hydrophobic octadecyl chains using the Surface functionalization technique, the release of the drug from the delivery system becomes quicker than SBA-1 [6].

Drugs of Biopharmaceutical classification system II (BCS-II) that followed the problem of low solubility, but high permeability was resolved using techniques including solid dispersion techniques, use of the drug in amorphous form, and complexation techniques. With an increase in solubility, the bioavailability of the longer half-life drugs also increased, which results in the accumulation of drugs leading to drug toxicity. Mesoporous silica materials (MSM) and other mesoporous particles exhibit hexagonal structure (MCM-41) having an increased surface area used as a reservoir for drug release. HMS and MCM-41 both were used as drug carriers for drugs that exhibit low solubility [7,8].

### 1.1. Development of Coating Processes

The concept of coating was initiated in ancient times. To begin with, Rhazes used psyllium seeds mucilage to unmask the taste of pills. Subsequently, it was reported that Avicenna coated the pills with silver and gold. In those days, various materials were used for coating purposes. Talc was introduced by White, known as a pearl coating and used to coat pills. In 1838, Garot introduced the coating of pills by using gelatin. Poisoned tablets were coated with wax to prevent unintentional poisoning. In earlier times, working individuals at pharmacies coated tablets for extemporaneous compounding only; later, this practice started in the pharmaceutical industry, and production was started on a large scale. In 1842, the first sugar-coated (SC) pill was imported from France to the United States (US). In 1856, a Pharmacist in Philadelphia pharmacist, indigenously manufactured coated pills [9]. By 1950, SC was considered the technique for coating purposes, and much work was done on that particular coating technique [10,11,12,13]. Firstly, coating in pharmaceutical industries was done on the pans, and the same technique was used for coating candies. The technique was further modified and evolved by the Middle Ages. Currently, pharmaceutical industries are well developed and organized using a wide variety of coating processes, including coating pans. The pans are of broad designs and range constructed of stainless steel. The coated material was then dried using heated air. Extra moisture and dust from the pans were removed using an air extraction system. By the twentieth century, conventional pharmaceutical processes of pan coating were employed for SC. Until the start of the 1950s, SC was the dominant type of coating in the pharmaceutical industry. Meanwhile, a new type of coating was introduced at that time, called film coating. Film coating (FC) gained significant interest because of its significant improvements in the possible limitations of SC processes. SC requires more operator skill, and it is a long operating process that kills time [14,15,16]. FC reduces the time, and less skilled operators, were required for that coating process [17]. Dissolving suitable polymers in volatile solvents could reduce the products’ coating duration from days to a few hours [9]. Moreover, FC improves the stability of dosage forms by protecting them from temperature, light, and humidity. FC also improves aesthetic properties and masks the unpleasant odor and taste [18]. Different types of drug release can be obtained by film coating, ranging from conventional to modified release, including enteric-coated, extended, and delayed-release [12].

The tablet is one of the most popular and extensively used solid dosage forms. Tablets are the compressed dosage form that may or may not contains the active ingredient. They differ in size, shape, and weight depending on the mode of administration and active ingredients used. Of all the dosage forms, approximately 70% of the medications were administered in tablets [15]. Tablets have some advantages over other dosage forms, such as feasibility, precise dose, and patient compliance, because they are produced on a large scale [19].

Patents appeared to be a source of innovation and a critical asset for any company. Patents provide detailed information about presenting, analyzing, and searching for scientific information. The search for patents has drastically changed from manual search to online search due to inventions in the field of computer science [20]. Some of the patents regarding FC are represented in Table 1.

### 1.2. Definition and Scope of Pharmaceutical Coating

The coating is defined as a procedure in which the desired dosage form may be a granule or tablet coated with an outer dry film to obtain specific objectives such as masking taste or protecting against environmental conditions. The coating material may be composed of coloring materials, flavorants, gums, resins, waxes, plasticizers, and a polyhydric alcohol. In the modern era, polymers and polysaccharides were principally used as coating materials along with other excipients like plasticizers and pigments. Many precautions must be considered during the coating process to make the coating durable and steady. According to the International Council for Harmonisation (ICH) guidelines, organic solvents are avoided in the formulation of pharmaceutical dosage forms due to their safety issues [21]. Tablets that are susceptible to degradation by moisture or oxidation must be coated by using the FC technique. This technique could increase its shelf life, mask its bitter taste, and make a smoother covering, which makes swallowing easier. Chitosan and other mucoadhesive polymers were also used for coating tablets to adhere these tablets to mucous membranes and achieve sustained drug release in localized areas [22]. In recent times, coating of the dosage form by using biopolymers has been extensively studied [23]. Active pharmaceutical ingredients (APIs), which are sensitive to light, can be protected by coating with opacifying agents. Similarly, enteric-coated tablets reach the intestine after an extended time and possibly help maintain the efficacy level of acid labile APIs [24].

### 1.3. Objective of Coating

Common forms of tablet coating are FC and SC. The coating helps maintain the physical and chemical integrity of the active ingredient; meanwhile, it also controls the drug release as it is controlled or continues to be released at a specific target site. Additionally, the coating was used to enhance the elegance of the pharmaceuticals, and the sophistication of appearance was enhanced by printing or making them with attractive colors [25].

### 1.4. Benefits of Coating

Coating provides stability to the tablets in handling and prevents them from sticking together. The coating also improves the mechanical strength of the dosage form, causes the dosage form smoother and more suitable for swallowing purposes. Pharmaceutical companies could print their marks, symbols, or abbreviations on the tablets and mask a disagreeable color or odor of the tablets. The release of the active ingredient can even be controlled with the help of coatings. Coated dosage forms could be site-specific. The coating prevents acid-sensitive drugs from having a negative impact on the intestine. The drug release rate in the gastrointestinal tract (GIT) could be controlled by controlling the dissolution rate of the tablet [26].

### 1.5. Drawbacks of FC

The drawbacks of FC are represented in Table 2 [27].

## 2. Film Coating

It is a process in which a thin coat of a polymer material is coated with oral solid dosage forms, including particles, granules, and tablets. Coating thickness may range from 20 to 100 μm [28].

### 2.1. Organic Film Coating

Based on the material used for the coating perspective, the binding material can be changed accordingly. Organic film coating may include water-based paints, lacquers, and enamel [29].

### 2.2. Aqueous Film Coating

The disadvantages of SC have led to the development of aqueous FC methods. Previously, these methods employed organic solvents, but due to the safety issue of these solvents, a better and more cost-effective method was developed in which the solvent was switched by aqueous-based FC [30]. These are applied as a thin film on the surface of the dosage form to obtain numerous benefits, including modified release, environmental protection, and taste masking. The coating depends on several factors, including tablet shape, the liquid used for coating purposes, equipment used for coating, and characteristics of the tablet surface. The coated film must be smooth in appearance, stuck smoothly with the tablet’s surface, and maintains physical and chemical stability. Based on the solubility of the water and the former film polymer used, the coating could be done by the solution or dispersion method [25] (Table 3).

## 3. The Present Trend of Aqueous FC in Pharmaceutical Oral Solid Dose Forms

Despite the purpose and rational use of FC techniques, the aqueous coating could possibly be reported as the most widely used method for coating purposes. Aqueous coatings are used for conventional and delayed-release systems [32] (Table 4).

### 3.1. Polymers Used in Pharmaceutical Coating

Polymers play a vital role in coating technology; sometimes, they are used for modifying the delivery of dosage forms, taste masking, and film forming agent. Some of the polymers used for such purposes are illustrated in Table 5.

#### 3.1.1. CAP

**Figure 1:**
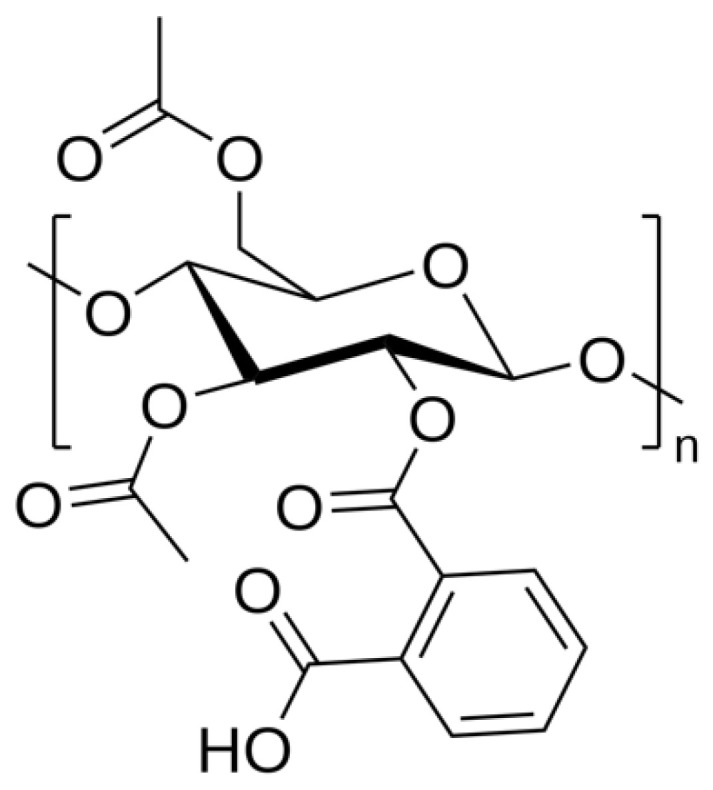
Represents the chemical structure of CAP [39].

To achieve enteric coating or controlled release of tablets or capsules CAP (the chemical structure shown in Figure 1), phthalate, Cellacefate, cellulose acetate, and cellulose esters were commonly used. To provide delayed action regarding drug absorption, CAP disintegrates at a pH greater than 6, producing it as a natural polymer used for enteric coating. Its properties determine that it is hygroscopic, which makes it vulnerable to solubility and penetration of moisture into GI fluid [37]. The molecular weight of CAP represents another parameter that affects the properties of the polymer. The properties of polymer vary with variations in the factors like viscosity, surface tension, conductivity, and rheology. A polymer with lower molecular weight yielded beads, while fibers with large diameters were yielded with a high molecular weight polymer. Polymers with high molecular weight were utilized for electrospinning to achieve formulation-based required viscosity. The viscosity of a solution directly reflects the chain entanglement of polymer chains. In contrast, processing electrospinning chain entanglement of the polymer depicts a vital role [38].

#### 3.1.2. Cellulose Acetate Trimellitate (CAT)

**Figure 2:**
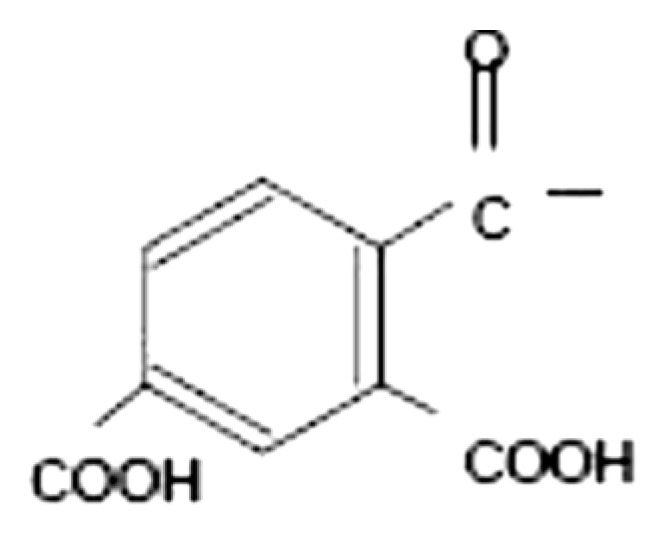
Represents the chemical structure of CAT [39].

Both CAP and CAT are similar other than the occurrence of the carboxylic group on the aromatic ring of CAT (as represented in Figure 2). Manufacturers quoted a value of 22% for acetyl and 29% for timellityl correspondingly. This polymer proves its enteric coating property by dissolving at pH 5.5 in the upper part of the intestine. Dissolution studies further demonstrated that both CAP and CAT exhibit similar solubility properties in organic solvents. Meanwhile, regarding aqueous solvents, studies have demonstrated that, while achieving full enteric properties, ammoniacal solutions of CAT were utilized with water. The plasticizers recommended to be used with aqueous solvents include acetylated monoglyceride, diethyl phthalate, and triacetin [40].

#### 3.1.3. Methylcellulose (MC)

**Figure 3:**
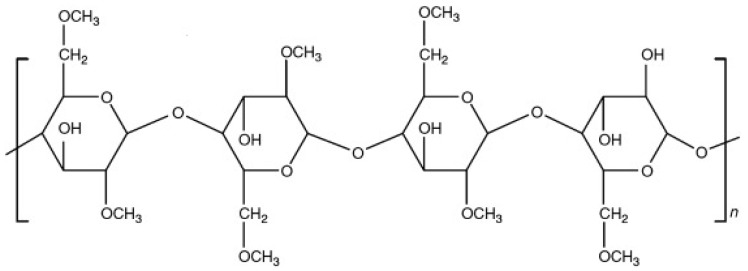
Represents the chemical structure of MC [42].

One of the most commonly and commercially used polymers is MC. The polymer is cellulose ether and has several industrial applications. It is the cellulose derivative with a structure comprising a methyl group followed by anhydrous-D-glucose moiety, which substitutes hydroxyl group (OH) at positions of C-2, 3, and 6 (as represented in Figure 3). One of the most important esters of the methyl family is methyl cellulose (MC). Structurally it consists of a methoxy group that accounts for approximately 27.5–31.5% of the whole MC. An aqueous solution of MC showed heat-related gelling properties. It is soluble in water. Its average molecular weight ranges between 10,000–220,000 daltons. It is most commonly used as a coating agent, binder, and disintegrant in oral solid formulations. Furthermore, it is also used for sustaining the drug release [41].

Polymer exhibits exceptional amphiphilic and physicochemical properties. Solubility of the polymer shifts from water-soluble towards organo-soluble, which depends upon the placement of the OH group upon its substitution from three to zero. Meanwhile, by increasing the temperature of the polymer towards a critical temperature, Singular thermal behavior was observed, which reduces the viscosity and produces an aqueous solution. With a constant rise in temperature, the lowest critical solution temperature (LCST) of the polymer MC was observed that produced a thermoreversible gel with augmented viscosity. Below LCST temperature MC is highly water soluble, while the polymer becomes insoluble at temperatures exceeding LCST. That could be the possible reason that the saturated solution of the polymer converts to a solid state upon heating [24].

#### 3.1.4. Ethylcellulose (EC)

**Figure 4:**
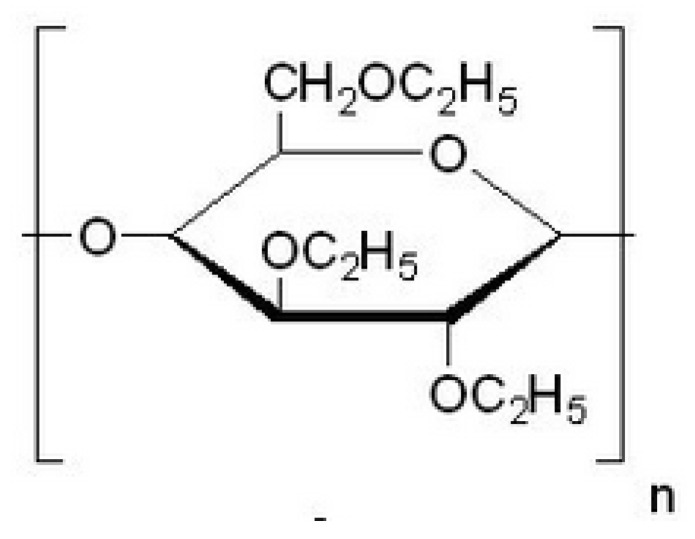
Represents the chemical structure of EC [46].

Directly, EC is water insoluble; it is further made water and fluid soluble after addition with other additives like HPMC (as represented in Figure 4). It is a partial derivative of cellulose ether (*O*-ethylated). EC is available in various molecular grades, which vary in viscosity. With the structural combination of alkali cellulose and ethyl chloride EC was prepared. The substitution of ethoxy groups was controlled throughout this reaction. In pharmaceutical formulations, EC is used as a binder, taste masking agent, and modified release agent [43].

The polymer is non-toxic, colorless, and tasteless and is widely used in organic solvents. EC can resist drug release. EC can also be used to incorporate materials by employing direct compression or wet granulation. Different microencapsulation techniques were used for the encapsulation of EC microparticles. It is one of the most widely used polymers for coating solid dosage forms that are water-insoluble [44]. Colorectal capecitabine-based microspheres were developed by Kumbhar et al. with the help of natural polysaccharide polymers to enhance cost-effectiveness. Microspheres were developed using single emulsification technology using calcium chloride (CaCl_2_) loaded with pectin, which was further coated with EC using the solvent evaporation technique. Furthermore, characterization of the microspheres was done, which includes particle size, Fourier-transform infrared spectroscopy (FTIR), surface electron microscopy (SEM), differential scanning calorimetry (DSC), drug release, and entrapment efficiency. Drug release studies observed that less than 20% of the drug was released in an acidic medium. An initial burst of drug release was observed, but at the end of the 12th hour, a total drug release of 85.33–95.55% was observed due to coating with EC. It was concluded that capecitabine-based microspheres loaded with pectin and coated with CE were also used effectively in the treatment of colon cancer and can replace conventional therapy [45].

#### 3.1.5. Hydroxyethyl Cellulose (HEC)

**Figure 5:**
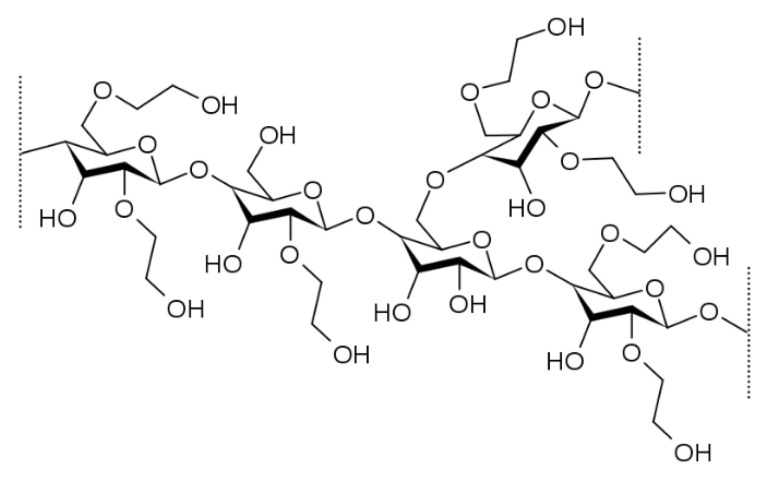
Represents the chemical structure of HEC [49].

It is a cellulose-based polymer used for gelling and thickening properties. HEC (the chemical structure represented in Figure 5) is further used in the hydrophilization process, which increases the solubility profiling of drugs within GI fluids. HEC has a molecular weight of 90 kDa, improved water solubility and neutral nature, making it an excellent candidate for drug carrier systems. Regarding its demand, its high biocompatibility, chemical stability, and exceptional thickening property make it a good candidate for pharmaceutical formulations. Before the formulation of a carrier system, the characteristics of both the drug and the carrier must be examined carefully [47].

It is further used as cleaning solutions, household products, and cosmetics due to its water-soluble and non-ionic abilities. HEC produces crystal-clear gels and solidifies the water phase of cosmetic emulsions. This polymer has a big disadvantage: it forms agglomerates or lumps when it first gets moistened with water. One of the grades of HEC, termed as R grade, is used for solution formation because no lumps were formed as it comes in contact with moisture and ultimately enhances solubility and processing time of the reaction [31]. Chowdary et al. developed a bilayer film-coated tablet of paliperidone. The tablet was further characterized for in vitro drug release studies. The tablet core was formulated with varying concentrations of polyox. An enteric coating optimized the coating with cellulose acetate and a sub-coating using HEC. Different influencing factors like the composition of tablet core and ingredients of the coating were investigated. The formulations were optimized by comparing the results of in vitro drug release studies [48].

#### 3.1.6. Hydroxypropyl Methylcellulose (HPMC)

**Figure 6:**
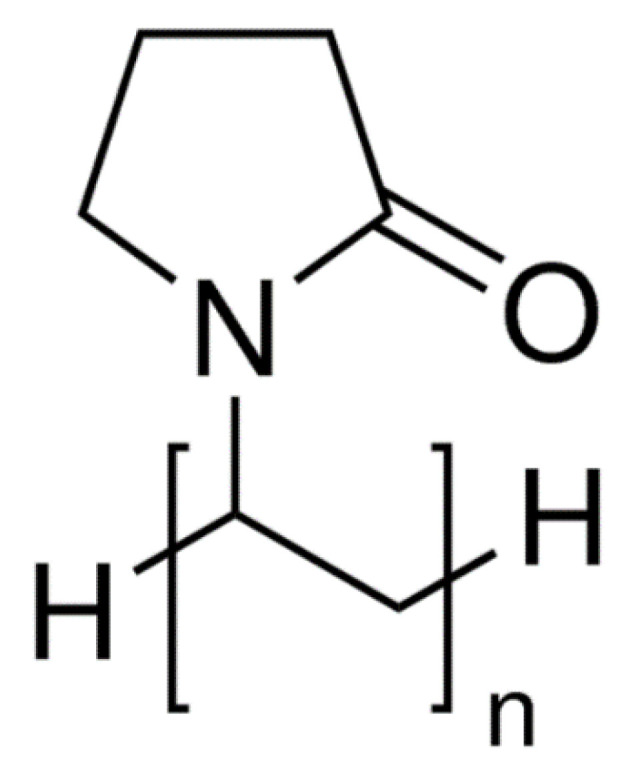
Represents the chemical structure of HPMC [52].

HPMC is a synthetic alteration of natural polymer (chemical structure shown in Figure 6). It is white to slightly off-white, odorless, and tasteless. It is a water-soluble polymer and can also be used in the controlled release delivery of tablets. It is also used for coated and uncoated matrix tablets. Upon hydration of the matrix with water, the polymeric chains disentangled [50]. Drug releases from drugs follow a two-way mechanism; in the first step, the drug is diffused from the gel layer of the polymer, while in the second mechanism, the release of the drug is followed by erosion of the swollen layer. As a result of the presence of cellulose ether, it is possibly used for the controlled release of oral drug delivery. HPMC can further be used for aqueous and solvent film coating. Matrix-based tablets could be developed using wet granulation or direct compression [51]. In another study conducted by Ifat Katzhendler et al., the release of naproxen and naproxen sodium was studied by varying the molecular weight of HPMC. It was concluded from the results that when used alone, naproxen decreases the drug’s solubility while naproxen sodium increases the system’s pH and ultimately increases drug loading; hence, drug release also increases [50].

#### 3.1.7. Polyvinyl Pyrrolidone (PVP)

**Figure 7:**
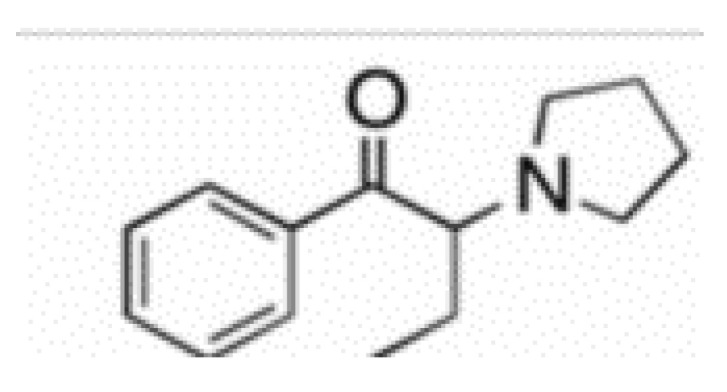
Represents the chemical structure of PVP [56].

It is a water-soluble polymer; its molecular weight ranges between 40,000 to 600,000 Daltons and can be distinguished into different grades. PVP is manufactured by polymerizing vinyl pyrrolidone in isopropyl alcohol or water (the chemical structure of which is represented in Figure 7). Due to the presence of the polar amide group and hydrophobic alkyl group, and the polar amide, it is highly water-soluble. Due to Its high degree of compatibility, it is an excellent candidate for a drug carrier system. PVP is a non-carcinogen, non-toxic, and temperature stable polymer. PVP exhibits a superior drug carrier system [53].

Different grades of PVP were used to enhance the bioavailability of poorly water-soluble drugs. In essence, it is used in tablet manufacturing as a binder. Granules produced by wet granulation using this polymer exhibit greater binding strength, low friability, and good flowability compared to other binders [54].

Tang et al. prepared paliperidone tablets using simple manufacturing and then coated them to produce a sustained effect. Tablets were evaluated and investigated for their in-vitro drug release behavior. Tablets were coated using a highly viscous HPMC K 100M and HPC coat. The in-vitro drug release parameters were evaluated considering different factors that include the core tablet composites, the material used for FC, and the formulation parameters. Gravimetric analysis was used to determine the drug release mechanism. The data obtained from drug release profiling were then put into the Peppas model. Drug releases at different intervals were then plotted in graphical form; the drug release was represented in the form of a slope at various time points. The results showed that the preparation could achieve better ascending drug release once the weight relation of paliperidone was 5:1 (core: layer). The fraction of HPMC and HPC was 33%. The ascending drug release was probably due to the penetration of solvent into the coated paliperidone tablets with the subsequent dissolution of the drug from the viscous polymer HPMC and HPC due to erosion of the matrix. Both erosion and diffusion mechanism of drug release was followed. It is concluded that coated tablets prepared by compression possibly are used for ascending control drug release over 24 h [55].

#### 3.1.8. Shellac

**Figure 8:**
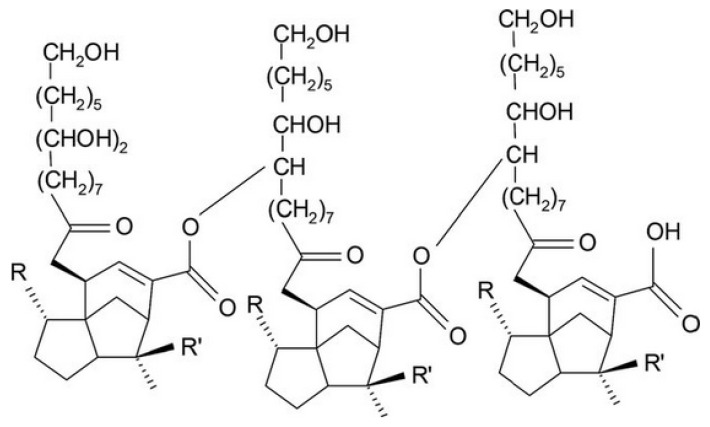
Represents the chemical structure of Shellac [59].

Due to structural novelty (as shown in Figure 8), shellac was considered to have unique properties. It is composed of an ester complex with polyhydroxy polybasic acids. Shellac has various applications, including adhesiveness, insulator, film forming agent, and thermoplastic agent. As shellac is obtained from animal origin and is completely different from other polymers, the unavailability of resins, aromatic compounds, resumes, phenolic compounds, oxidized polyterpenic acids, and resinotannols give it unique properties [57].

Shellac consists of an acidic group with a high acidic dissociation value. Due to this, it is not easy for the group to dissociate in a gastric environment, which causes a decreased dissolution effect in the stomach (pH 2). With the modification of shellac chemical structure with the addition of sodium carbonate (alkaline group) performance of shellac in the stomach was enhanced. In one study, nanoparticles and nanofibers of ketoprofen were formulated, incorporated with shellac, and done with its characterization (SEM, XRD, FTIR [58]). Results showed that nanocomposites were suitable for the controlled release of ketoprofen [59].

#### 3.1.9. Sodium Carboxymethylcellulose (SCMC)

**Figure 9:**
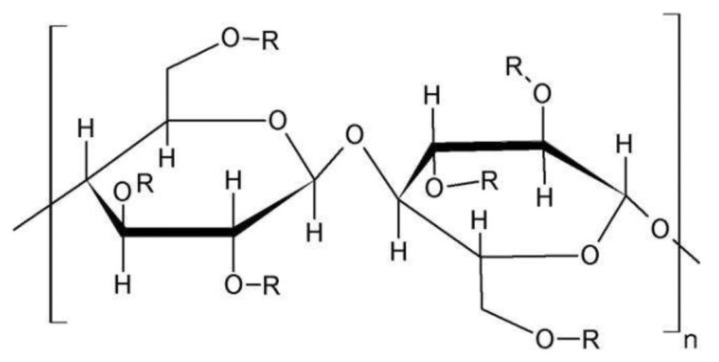
Represents the chemical structure of SCMC) [62].

It has a further polynomial cross-linked form known as croscarmellose sodium (Figure 9). It has excellent swelling properties, hydrophilic with excellent absorbing properties. Commercially SCMC is available with varying degrees of substitution (DS) ranging from 0.7 to 1.2, with a subsequent amount of sodium content of 6.5–12% of total weight. SCMC is extremely hygroscopic in nature and absorbs more than 50% of water content. Tablets formulated by using SCMC tend to harden with time [43].

Croscarmellose sodium enhances the bioavailability of numerous formulations, giving excellent disintegration and dissolution characteristics. In oral formulations, croscarmellose sodium is used as a disintegrant. While related to the pharmaceutical industry, it is used to develop tablets with direct compassion and as an insecticide employed in the paper and textile industries. It behaves as a protective colloid to prevent water loss [60]. Shinde et al. tried to develop sustained swellable matrix release tablets using diltiazem hydrochloride as a model drug. The purpose of the dosage form was to improve the dissolution profile of the drug as the drug is more soluble in the upper part of the GI tract [61].

#### 3.1.10. Zein

**Figure 10:**
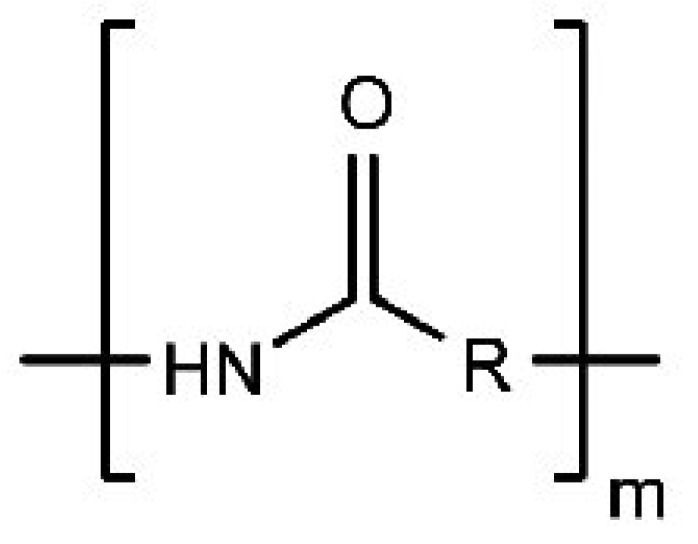
Represents the chemical structure of Zein [65].

It is a natural polymer derived from plant origin and is more beneficial than synthetic polymers. It has applications for controlled drug release and biomedical purposes. Zein is highly nutritive due to the presence of numerous components, which include proteins. It comprises 50% corn protein and 6 to 12% protein according to its dry weight. About 25% of this protein is present between the bran and germ, while 75% of this protein is present in endosperm tissues. Zein is also used in vaccines, tissue engineering, and gene delivery. It is used as a biopolymer due to its two basic properties: biodegradability and biocompatibility [23]. A complete illustration of zein structure was not discovered until now, but with the help of chromatographic techniques, some of its characteristics were discovered in the 80s. With the help of the small-angle X-ray scattering (SAXS) technique, the helical structure (with ten successive folds) of zein was revealed [63]. Zein was obtained in α-, β-, δ-, and γ forms depending on the molecular weight and extraction method used. It was further used in various industrial fields, including adhesives, ink, food industry, ceramics, ink, chewing gums, candy formation, plastic packaging materials, and adhesives (Figure 10). Initially, zein was used as protective material on coated materials because it is more resistant to humidity, abrasion, and heat tolerable. Due to its low cost, it was also used as a taste-enhancing agent in an immediate release dosage form. It was concluded from a study that there appeared to be no influence of the coating process on the hardness of the core. However, tablets coated with zein (FC) showed a high strength compared to HPMC and CAP [63].

Zein exhibits excellent physical characteristics, which is why it is used in different formulations, including gels, fibers, films, nanoparticles, and for the controlled release of drugs in tablets. Products prepared using zein have improved shelf life because zein is resistant to water, heat, and abrasion [40]. Van et al. inspected zein as a coating material by preparing prednisolone for colon-specific drug delivery. A suitable proportion of zein and Kollicoat MAE 100P were prepared and tested to confirm the strengthening capacity of zein films. It becomes evident from the specific dosage form of the colon that zein exhibited an immediate release of the drug substance immediately as it reaches the basic medium of the intestine. Furthermore, the formulations were characterized by FTIR, and it was evident that different ratios of zein and Kollicoat MAE 100P experience physical interactions [64].

#### 3.1.11. Eudragit L-100-55

**Figure 11:**
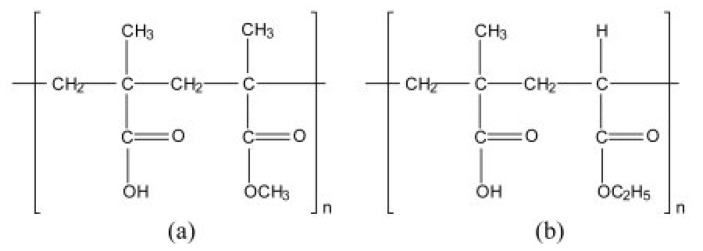
Represents the chemical structure of (**a**) Eudragit L 100 (**b**) Eudragit L 55 [69].

It is a copolymer obtained from the esters of methacrylic acid and acrylic acid, where the functional group (R) is responsible for its physicochemical properties (chemical structure represented in Figure 11). Eudragit is anionic, white in color, and has free-flowing properties. It is used for entering coating purposes and dissolves at a pH of 5.5 or more [66]. One of the pharmaceutical industry’s most commonly used pH-sensitive polymers is Eudragit because of their soluble nature at various pH ranges. At a pH higher than 5.5, Eudragit L100-55 controlled the release of the pharmaceutically active ingredient. There appeared a difference in Eudragit L100 and Eudragit L100-55 by substituting a methyl group rather than an ethyl group. The difference in the functional groups eventually imparts a change in the dissolution profile of both polymers at different pH values [67].

Alsulays et al. developed enteric coated tablets of lansoprazole to improve their physical and chemical properties by using a new technique named hot-melt extrusion. Kollidon 12PF was used as polymer, Lutrol F68 was used as a plasticizer, and magnesium oxide (MgO) as an alkalizing agent. An amorphous state of lansoprazole appeared and presented a better drug release when it was extruded with Kollidon 12 and Lutrol F68. At the same time, incorporating MgO improved the extrudability of lansoprazole and its release, resulting in more than 80% of drug release within the buffer zone [68] (Figure 11).

#### 3.1.12. Other Additives

##### Plasticizer

These are low molecular materials that were added to enhance the mechanical strength of a polymer [70]. Plasticizers weaken the intermolecular forces of the polymers, thereby reducing their rigidity and improving their coalescence properties while making films [70]. They can reduce the glass transition temperature of amorphous polymers, decrease the interactions of different polymers, and reduce the brittleness of films [70]. They alter the plasticity of film-forming polymers (FFP) in two basic ways, external and internal plasticizing. External plasticizing involves the use of plasticizers, while internal plasticizing appears to be due to a modification in chemical structure that ultimately changes its physical properties. External or internal plasticizers were used in an optimum range, which ranges from 1–50%, but most commonly 10% plasticizers were used. Polyethylene glycol and HPMC were the polymers most commonly and effectively used. Triacetin, a less commonly used plasticizer, protects the aqueous coating by creating a moisture barrier against the coat and protects the formulation [12].

##### Colorants and Opacifiers

To improve product identification, enhance the appearance of products, and decrease the risk of counterfeit products, colorants were added to the formulations. Opacifiers were used in those products that were damaged by light. The ideal concentration of colorants used in film coating formulations (FCF) ranges from more than 2% *w*/*w* for dark shade and 0.01% *w*/*w* for light shade. Each country has its own regulatory approved opacifiers and colorants. Some of them are mentioned in Table 4. Colorants may be water-insoluble, known as pigments, and water-soluble colorants, known as dyes, as represented in Table 6 [12].

### 3.2. Issues Related to Aqueous Film Coating

The FC process must be treated at a temperature above the polymer’s Tg. Additionally, the quantity and quality of the pigment and plasticizer in the coating process influence many of the mechanical properties, barrier properties, physicochemical properties, and other factors discussed in Table 7 [12].

### 3.3. Equipment Used for Tablet Coating

Equipment was generally used to coat the tablet surface with a thin film that acts as a coating material. The general purpose of the film was to prevent the tablet from physical or chemical harm and mask the unpleasant smell, odor, and taste. The coating also protects the tablet from the harsh gastric environment and promotes sustained drug release. The coating also enhances the appearance of the tablet [71].

Equipment used for coating purposes was constructed on simple principles: the coating is applied on the tablets in a solution form while the rotator is moving horizontally or vertically. During rotation, a stream of hot air is also introduced, which promotes the evaporation of the solvent. Continued movement of the beds causes an even distribution of the coating material over the tablets and even drying [26].

Some of the important parameters of the coating process are as follows:

### 3.4. Configuration of Coating Material

Coating material usually consists of a solvent carrier system and the dissolved coating material meant to be coated on the tablets. The solvent carrier system evaporated with the help of the drying mechanism during the film coating process. The heat was delivered with the inlet air used to evaporate water, while the exhausted air appeared to contain more water content due to the evaporation process. Thus, the exhausted air became cooler in temperature compared to the inlet air until the entire drying process was completed.

#### 3.4.1. Capacity of Air

It represents the amount of solvent or water removed during the coating process. It depends on the rate and extent of air that flows through the bed of the tablets.

#### 3.4.2. Efficiency of the Equipment Used

The coating material’s adherence to the coating pan’s walls will determine its efficiency. In the case of sugar coating, the efficiency is very less, while the satisfactory limit of equipment efficiency reaches 60%.

#### 3.4.3. Surface Area of the Tablets

The coating parameters were affected by the tablet surface area and size. The smaller the tablet size, the larger the surface area per unit weight [31].

##### Standard Coating Pan

**Figure 12:**
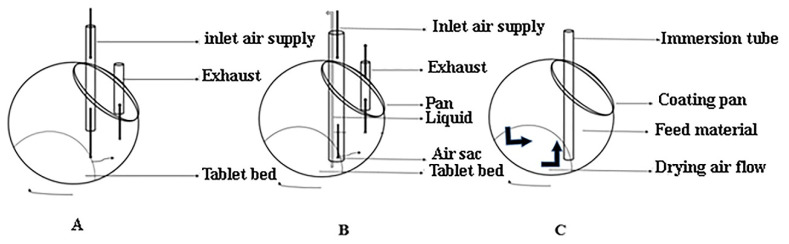
Shows (**A**) Standard coating pan, (**B**) Immersion tube system, and (**C**) Glatt immersion sword system.

Conventional pan systems or standard coating pans, which are similar, were mostly employed by pharmaceutical industries. Specifically, they were designed for coating purposes in such a manner that the circular pan is considered a drum, which is metallic and has a diameter of 6–80 inches. The drum was further tilted from the top of the bench at an angle of approximately 45 degrees. An electric motor was fitted in standard coating pans that rotated the drum on its horizontal axis, which tumbles the tablet’s batch. It is a fast process and decreases the drying time. These conventional coating pans can further be used for sugar or film coating purposes with slight modifications, which include the use of an immersion sword, pellegrini baffled pan, pellegrini baffled diffuser, and immersion sword, as illustrated in Figure 12. On the contrary, the equipment has some disadvantages, including using organic solvents, which might be risky, and air supply, if unregulated, can complicate the process. Furthermore, as drying occurs on the surface of the tablets only, this might lead to improper coating and mixing of the tablets [31].

##### Immersion Sword

It is a technique used to increase the drying productivity of a conventional pan coating apparatus. In this process, a perforated metal sword is inserted into the bed of tablets. Due to the presence of a perforated sword, this system allows the circulation of just one flow of dry air through the middle portion of the sword and resists many flow points of air.

##### Immersion Tube System

The commercially available immersion tube system consists of an additional tube further immersed in the bed of the tablet coating machine. The function of the tube-nozzle was to provide both the coating solution and hot air concurrently. It is a long tube with a spray nozzle at its tip. It was designed in such a manner that heated air leaves the system by flowing in an upwards direction through conventional ducts. The drying time and efficiency of a standard coating pan could be enhanced with the simple inclusion of an immersion tube system. This technique may be used for film and sugar coating [10].

##### Baffled Diffuser and Pan

The drying efficiency of standard pans used for coating purposes was improved using the Pellegrini baffled diffuser and pan technique. One of the possible reasons was that they improved the drying and tumbling of the coating equipment. The tablet coater was successfully used to evenly distribute the drying air over all coated tablets. Ordinary coating pans with baffling diffuser and pan were only suitable for sugar coating purposes due to drying capacity limitations [46].

#### 3.4.4. Perforated Coating Pan

**Figure 13:**
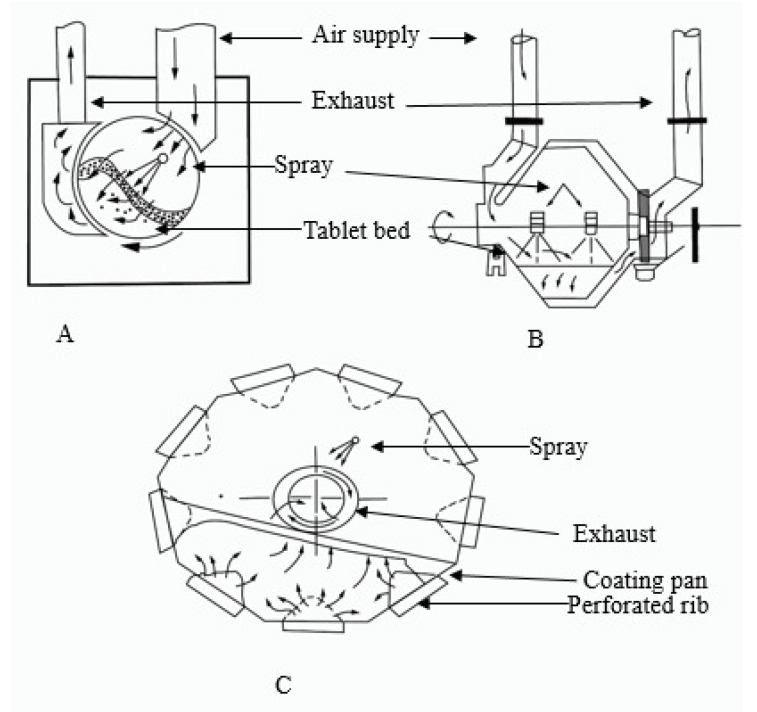
Represents (**A**) Acela coat system, (**B**) Hi coater system, and (**C**) Dria coater system.

Among other coating techniques, many pharmaceutical companies widely adopted perforated coating pans. These coating pans consist of a full, partial, or one perforated drum. Like other pans, the drum of this coating pan rotates on its horizontal axis. The perforated drum is on the horizontal axis and equipped with an air-atomized spray nozzle and airflow controller. Perforated coating pans have an efficient drying mechanism; unlike other coating pans, perforated pan coaters have an effective drying system, as illustrated in Figure 13. Moreover, they have a high capability in the tablet coating process. They are used for both sugar coating and aqueous film coating. Perforated coating pans appeared to be efficient for film and sugar coating, compared to conventional pans, due to their high coating capacity, numerous airflow patterns, and increased tablet drying [47].

##### AccelaCota System

In this coating system, hot air is passed directly from the top part of the drum which falls directly on the bed of the tablets, and the air is exhausted from the drum from the perforations present at the bottom of the drum. The material coated on the tablets is evenly distributed in the drum through spraying nozzles. Meanwhile, the presence of baffles in the drum improves the tumbling of the tablets and provides free mixing. It is used effectively for both coating (FC, SC) and drying processes [10].

##### Dria Coater Pan

This type of coating pan has perforated ribs that are present on the inner periphery of the coating drum. The working principle of Dria coater is like an Accela coating machine. Meanwhile, the air used for drying purposes enters from below the coating drum and flows through the tablets in an upward direction, eventually leaving the system from the back of the tablet coating pan [31].

##### Glatt Coater

One of the most advanced technologies, having a shorter processing time and higher coating capacity, is known as Glatt coater. It is designed so that one can easily direct the drying air inside the tablet coating drum. Generally, it consists of an exhaust system, and air after passes over the tablet bed exit from it. It has a unique design that reduces the turbulence produced by the spray nozzle, which ultimately ensures a smooth coating on the surface of the tablets. Furthermore, the pan is fitted with baffles, which protect the tablets from damage during mixing and enhance their mixing simultaneously. Glatt coater was also constructed with a perforated system like other coating pans. Spray nozzles were situated at the top of the drum while aiming toward the tablet bed and atomizing the fluid used for coating purposes [72].

##### Fluidized-Bed Coater

**Figure 14:**
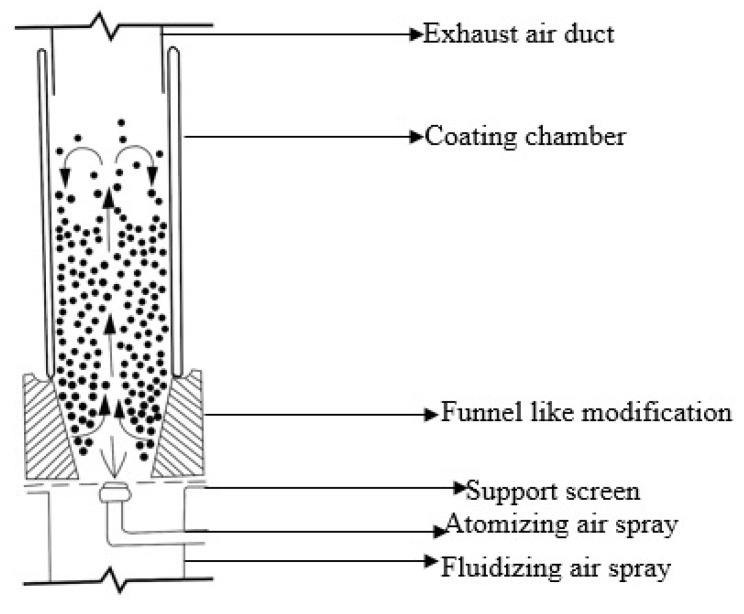
Represents fluidized bed coating system.

The coating mechanism in these coaters follows the fluidization principle; in these coaters, an increased amount of air enters through the center of the column, which raises the tablet in the center and proceeds the coating process. The fluid used for coating purposes is sprayed using spray nozzles placed at the top or bottom of the equipment [73]. It has a similar working mechanism to other bed coaters. It consists of a vertical cylinder in which the tablets are suspended in the chamber and dried due to an upthrust of drying air. A fluidization process will occur, which causes the tablets to move outward, upward, and then downward. The spray nozzle was then used to spray the desired fluid used for coating the tablets, either from the bottom or top of the fluidized bed coater, as shown in Figure 14 [74].

##### High-Pressure Airless Systems

It is used to pump out the liquid without the need for air at a very high pressure of about 250,300 psig. The nozzle used for this process is very small in size, about 0.0090 to 02 inches. The spray rate and degree of atomization depend on the liquid’s orifice size, fluid pressure, and viscosity. The size of the orifice and the pressure of the fluid are the controllers for regulating the degree of atomization and the spray rate [31].

##### Low-Pressure Air-Atomized System

This system uses a low pressure of 550 psig to pump the fluid through a 0.020-inch larger orifice. Some major parameters that regulate the spray rate and the atomization process are the fluid cap orifice, the pressure of the air, the design of the air cap, and the viscosity of the fluid [75].

##### Evaluation Parameters of FC Tablets

Hardness and Friability

Hardness and friability tests were conducted to ensure that the tablets’ mechanical strength persists during handling, transportation, storage, and usage. The hardness of the prepared tablets was performed using a manual or automatic hardness tester, and its units are in Kg/cm^2^. The friability of the formulated tablets was determined using a friabilator. The apparatus consists of a plastic body in which the tablets were rotated at 25 rpm and given a shock and abrasion condition from a height of 6 inches. The weight of the tablets before and after the experiment was determined. The friability of the tablets was determined by using Equation (1).

(1) $$\%\;\mathrm{Friability}=\frac{W1-W2}{W1}*100$$

Here, W1 represents the original weight of tablets, and W2 represents the weight of tablets after the experiment is completed. The friability value must not be greater than 1% [76].

Uniformity of weight

Uniformity of dosage form represents the even distribution of drug substances or excipients in all dosage units. The addition of the ingredients (active and excipients) must be within the range as claimed on the label. Content uniformity and weight variation were both parameters to determine uniformity in dosage units [77].

Disintegration time

According to pharmacopeial recommendations, one of the vital evaluation parameters for all capsules, granules, and tablets is the disintegration test. This specific test evaluates the performance and quality of a dosage form to disintegrate completely over time. For instance, if a tablet is highly compressed or the gelatin-based capsule does not obey pharmacopeial recommendations, then the time of dosage forms to disintegrate elevates. This test also ensures the consistency and uniformity of the contents within all batches. In case of any variation or if any sample does not comply with the result, suitable actions must be taken according to the results [31]. Disintegration tests were carried out in a disintegration apparatus recommended according to USP guidelines. One dose unit was introduced at a time. Temperature conditions and rpm were maintained accordingly [78].

In vitro dissolution studies and release kinetics

In vitro dissolution and release kinetics were evaluated to determine the amount of drug release from the dosage form. The amount of API released from a dosage form will ensure the presence of an active drug present for absorption at the site of action. As dissolution is directly related to bioavailability, increased dissolution ensures increased bioavailability of API. Mathematical models were used to investigate the drug release process. The system’s goal was to maintain the number of therapeutic moieties with therapeutic concentrations in the desired organ or blood. These mathematical tools better explain the release of APIs concerning time. Kinetic tools were used to evaluate the design of pharmaceutical dosage forms both in-vitro and in-vivo.

Stability testing

Stability studies of pharmaceutical formulations were conducted to ensure the formulation’s efficacy, safety, and quality. Stability testing was accelerated for 6 to 12 months, and additional tests were performed for 3 months while the product was stored at 50 °C with 75% relative humidity (RH) [31]. Stability studies ensure that the finished product bears the temperature variations produced from the manufacturing process to the use of the patient.

### 3.5. Pharmaceutical Application of Film Coating

#### 3.5.1. Modified Drug Release

In most cases, to achieve patient compliance or to improve drug efficacy, modified drug release systems were used [79]. Consequently, the tablets were coated as FC using suitable polymers that retard or control the drug release. Some of the approaches for a modified drug delivery system are as follows.

#### 3.5.2. Delayed Drug Release

One of the major advantages of EC is to increase the gastric stability of the dosage by protecting it from the harsh gastric environment. Polymers having pH dependency and solubility were mostly used for EC. They also tend to prevent the premature release of drugs in the stomach. Some of the drugs, which include proton pump inhibitors (omeprazole, esomeprazole, lansoprazole, rabeprazole, and pantoprazole), were acid labile and needed EC to prevent degradation in the stomach and ensure proper drug release [56]. Likewise, Gobinath et al. [80] formulated CE tablets using pantoprazole as a model drug using Eudragit and CAP. Tirpude and Puranik [81] proved that rabeprazole’s performance improves by using EC with two different enteric polymers: an outer coating with cellulose and an inner coating with acrylic polymer [81]. The enteric coating of the granules was also used to formulate a time-dependent drug delivery system to release APIs at different times, one of which dissolves in the upper and the other in the lower portion of the intestine. The Food and Drug Administration (FDA) has now officially accepted a formulation of dexlansoprazole that is formulated using two different types of enteric-coated granules that have different dissolution profiles related to different pH, one of which releases after 1–2 h of administration while the other after 5–6 h [82]. Using such formulations in once-daily dosing controls gastric acid contents for a longer time and prolongs drug absorption [82]. Macromolecules, including proteins and peptides, have low permeability and stability when administered orally. Thus, the enteric coating of the formulations was considered to overcome such issues and enhances drug release [83]. Wong et al. [84] prepared oral tablets by using insulin as a model drug, and the tablet was then enteric coated by using cellulose acetate hydrogen phthalate, and other additives, which include absorption enhancer (chitosan) and enzyme inhibitor (sodium glycocholate). This tablet showed maximum drug release at insulin-dependent Glut-4 translocation and decreased or no drug release at acidic pH [84]. Likewise, many other formulations for oral administration including hormones or insulin were considered or are present in the market [83,85].

#### 3.5.3. Colon-Targeted Drug Release

A colon-specific drug delivery system was used to treat numerous diseases, including irritable bowel syndrome (IBS), Crohn’s disease, and colon cancer [86,87,88]. Such a colon-specific delivery system could be used to administer proteins and peptides through this route, and their bioavailability could be enhanced [88]. Pathological conditions, motility, pH, and fluid content of the GI tract change from the colon, so the materials used for coating purposes are more complex than in oral dosage form [87,88,89]. Ibekwe et al. [90] developed a new colon-based system triggered by bacteria and pH-dependent in a single-layer matrix film. To facilitate a site-specific delivery system, prepared tablets were coated using a pH-responsive polymer [91]. Dodoo et al. [92] also developed probiotics and then coated them to find their effectiveness when given through the colon. Goyanes et al. [93] formulated budesonide-based colonic tablets for the controlled release of the active drug. The tablets were formulated in capsule form, while each capsule consisted of 9 mg of the active drug. The tablets were coated with Eudragit L100 and fabricated using 3D printing technology. They were further evaluated by scanning electron microscope (SEM) to investigate the outer coating. Drug release profiling was also done to ensure the release behavior of these tablets. The release of the active drug starts in the small intestine after 1 h after dosing, and the process continues sustainably under the circumstances of the colon and distal intestine [93].

#### 3.5.4. Chronotherapeutic Drug Release

The release of APIs could be programmed or delayed for a specific period to meet the needs of chronotherapeutic, particularly for the symptoms of circadian rhythms [94,95]. Diseases that include bronchial asthma, cardiovascular disease, sleep disorders, and rheumatoid arthritis that are likely to appear in the early morning or night are the best examples of circadian rhythms. The enteric coating was also used to achieve chronotherapeutic drug release. Luo et al. formulated a combination of fixed-dose by using pravastatin sodium and telmisartan in a tablet coated with an enteric coating that matches with circadian rhythms of cholesterol and hypertension and cholesterol and is administered before bed once daily [64]. Enteric coating prevents the early release of the drug from the tablet at acidic pH in the stomach, but it finally releases the drug at pH 6.8. Similarly, a delivery system that provides delayed release of therapeutic moieties at bedtime dosage treatment is therapeutically suggested, matching the variation in blood pressure and cholesterol synthesis due to circadian rhythmic variations. This system has the benefit of providing maximum therapeutic effect [90].

#### 3.5.5. Sustained Drug Release

The amount of drug release could be controlled by using the amount of polymer during surface coating. Tortuosity, the permeability of coating membrane, and thickness, so, by altering these factors, different releases of the drug could be achieved. To achieve sustained drug release, the coating materials are pH-independent and water-soluble [90]. Optimize drug release has also been attempted using hydrophilic and hydrophobic polymers in combination. A commonly used antidepressant, venlafaxine HCl, has a very short half-life of about 5 h, so to reduce its dosing frequency, a sustained release formulation was developed. Jain et al. [96] formulated an organic and aqueous-based reservoir type coated tablet using venlafaxine as a model drug for sustained drug release. In such, a formulation polyacrylate was used as a coating agent while ethyl cellulose was used as dispersion. Wan et al. [97] prepared loxoprofen sodium-based sustained release pellets via double-layered coating. These pellets consist of a dissolution rate that regulates the sublayer with HPMC and pH modifier (citric acid) and an external distribution rate that regulates the coating using EC as aqueous dispersion on the surface of the loaded pellets with drug [97].

#### 3.5.6. Taste Masking

In the case of geriatric and pediatric patients, one of the major hurdles in medication intake is unpleasant taste. Bitterness is the main cause of medication repletion. Thus, one of the key parameters to improve patient compliance is to mask the unpleasant taste [98]. Meanwhile, masking the taste must not mark any negative effect on the dosage form, including affecting the bioavailability of the drug, causing irritation of mucosa, dryness of mouth, or obstructing swallowing [99]. Different methods were employed for taste masking, including surface coating, the addition of flavoring agents, complexation, salt formation, and chemical modification [98]. Amongst all these methods, one most effective and commonly used method is FC [98,99]. Many synthetic and natural polymers are available that are used for taste masking. Hydrophilic polymers include derivatives of starch, e.g., cellulose ethers, hydrophilic block copolymers, and starch derivatives, as well as gel-forming and lipophilic polymers were also used for the masking of taste [100]. Polymers may be used in combination or alone. Commonly, they are used in combination with hydrophilic and hydrophobic polymers in different concentrations [98,100]. In a study conducted by Nishiyama et al. [101], FC was done to mask the unpleasant taste of lafutidine. Orally disintegrating tablets were prepared using water-soluble and water-insoluble polymer (Hypromellose and ethyl cellulose). The polymer ratio affected the tablets, including their tensile strength, drug release, lag time, and water permeability [101].

#### 3.5.7. Active Film Coating

It is a process of coating tablets or granules that contain APIs using a solution or suspension. The coating was done to improve product stability, prevent any interaction between APIs, and the development of a fixed dose combination [102,103]. Hydrophilic drugs easily dissolve in a solution or water-based coating suspension and then easily be sprayed on core tablets. Hence, the coating process is easier for hydrophilic drugs than lipophilic drugs [102]. Moreover, to protect spray nozzles from powder clogging, the particle size of water-insoluble drugs must be very small. Meanwhile, the coating process must be homogeneous to obtain acceptable uniformity of content [78]. There appeared some challenges in the active coating, which include [104] determining the end point of coating attaining targeted potency[88], confirming weight variation in each tablet[105], and maximizing the efficiency of the coating process[102,106,107]. During the FC process, random tablets were selected and weighed to ensure any weight gain and the quantity of APIs deposited on the core of the tablets during the process assay [103]. Based on this assay, further quantities of coatings suspension or solution were added to attain the desired potency. A linear relationship was observed between the coating time and the number of APIs to be deposited when the coating conditions, particularly the spray rate, remained constant [102,106,107]. The uniformity of the contents could possibly be affected by various factors which include the temperature of air, the rate of spray, the speed of the pan, the residual moisture and the atomization pressure [106]. Thus, it is significant to realize the factors in the coating process that affect content uniformity [102].

#### 3.5.8. FC in the Field of Nanotechnology

Researchers have struggled to formulate and optimize magnetic nanoparticles in recent years, which appeared to be helpful in biotechnology, computer, and drug delivery. The application and performance of such dosage form are highly influenced by its proper synthesis and design. Until now, many nanoparticles using metals such as copper, iron, magnesium, manganese, and their oxides have been developed effectively. Some conditions, which include coating surface, shape, particle size, surface charge, and magnetic properties of the particles were effectively monitored during the synthesis process. After choosing a suitable method for synthesis, the shape, size, colloidal stability, and surface coating of the nanoparticles were controlled in the optimum range. The efficiency of the coating process depends on the coating system (especially its mechanical properties), concentration and type of the suspended material, and the treatment of the metal surface before the conduction of the process. Generally, the coating solution consists of additives, a pigment, a filler, and a binder. Ideal coatings possess better stability, low permeability, and cost-effectiveness [108] (Ansari, Kadhim, Hussein, Lafta, & Kianfar, 2022).

#### 3.5.9. Marketed Available Products

Some of the marketed available FC products are presented in Table 8.

## 4. Conclusions

It was found that tablets were the most common and ancient dosage form. Before the invention of proper machines for their manufacturing, tablets were made with the help of hands. Thus, to mask the unpleasant taste of different active constituents, to prevent them from atmospheric conditions, or to prevent a harsh gastric environment, coatings were done. Different coating techniques were employed for the coating of dosage form, and each coating technique has advantages and disadvantages. FC is a critical but common process that provides a dosage form with different functionalities, thereby meeting diverse therapeutic needs. FC was rendered as the most suitable and weightless coating material. In the pharmaceutical industry, FC not only masks the unpleasant taste and increases patient compliance, but it also protects the APIs from direct contact with water and thus enhances their stability.

## 5. Current Limitations and Potential Challenges in the Field of FC

As FC appeared to be associated with some challenges addressed as follows.

- Due to the coating of the dosage forms, the processing time could be increased. The issue could be minimized by using a solid aqueous coating method.
- Water, used as a universal solvent, if not removed effectively, may initiate chemical reactions. However, some modern formulation procedures use solid coating methods to resolve such issues effectively.
- It is also possible that if the harsher process for coating or removal of water contents were used, they might affect the dissolution rate of the formulated dosage form. Thus, specialized coating formulations with specific pressure and temperature requirements were used to minimize such issues [110].

## Figures and Tables

**Table 1:** Representing some patents regarding FC.

Serial No	Patent Name	Patent No
1	Film coating composition	JP-5936732-B1
2	Film coatings and film coating compositions based on polyvinyl alcohol	EP1208143B1
3	Film coatings and film coating compositions based on dextrin	US6348090B1

**Table 2:** Representing drawbacks of FC.

Flaw	Definition	Possible Reason	Treatment
Blistering	Blistering refers to the detachment of film from the surface of the object (like a tablet) and results in the formation of blisters.	The possible reason for this defect could be the entrapment of gases in the layer of the film during the process of spraying (mainly when the process is overheated)	That defect could be treated by designing the drying conditions to be mild.
Chipping	Chipping states a condition where the film becomes dented, chipped from the edges.	Possibly due to a decreased rotation of the drum or flow of fluidizing air in the coating pan.	The operator must be careful at the pre-heating stage and not over-dry the tablets. Otherwise, the tablets encourage the defect by becoming brittle.
Picking	It is defined as the adhered film on the tablet’s surface that may be torn away, resulting in the sticking of tablets.	The main cause of such defect is the production of wet tablets, which may stick together.	The condition may be treated by reducing the volume of applied liquid or by increasing the temperature of dry air.
Pitting	In this type of defect, specific pits have appeared on the surface of the dosage form without any visual disappearance of the FC.	The reason for such a problem appeared due to the melting point of the materials used is less than the temperature of the tablet core used in the tablet formulation.	Adjusting the temperature during the process of tablet core results in the removal of such defects.
Roughness/Orange peel	It is a surface defect in which the film appeared to be non-glossy and resembled an orange	Insufficient dispersion of the coating solution before drying	The problem may be corrected by an additional solvent which causes the thinning of the solution
Color variation	By definition, color variation is a defect that results in the variation of color of the film	Duration of tablet appearance and variation in the spray zone’s frequency and the spray zone’s shape and size were responsible for such defects.	To solve such instability caused by the ingredients, a reformation with different additives and plasticizers is the best way to solve the problem.

**Table 3:** Characteristics between SC and FC [31].

Type	Properties	FC	SC
Process	Need for the training of the operator	The process is automated in nature, requiring the operator’s training	Substantial
	Conformity with Good Manufacturing Practices (GMP).	High	Difficulties may arise
	Process levels	It is a single-stage process	Multiple steps process
	Functional Coatings	Easily adjusted for modified release	Nil
Tablet	Appearance	They retain their original core	Usually round in shape.
	Change in weight after coating	Approximately 2–3% increase in weight appeared; they are not as shiny as SC	The weight increased from 30 to 50%
	Logos or break lines	Feasible	Not feasible

**Table 4:** Factors that affect the quality of film coating [31].

Factors Affecting the Quality of Film Coating	Factors that Affect the Coating with the Interaction of Substrate
Drying process	The viscosity of the coating liquid influences the coalescence of droplets
Interaction between core and coating material	There exists an influence of solid contact on the viscosity of the coating and the roughness of dry coating.
Uniform distribution of coating	There appeared a great influence of surface tension on the spreading of coating material across the surface of coated material, wetting the surface of the substrate, and evenly distributing the liquid in the form of thin film over the substrate

**Table 5:** Showed the polymers used for modifying drug delivery systems.

Polymer	Purpose	Reference
Ethyl Cellulose	Sustained release	[33,34]
Eudragit RS 30 D alone and in combination with ammonia methacrylate	Sustained release	
Eudragit NM 30 D and Eudragit NE 30 D in combination with ethyl acrylate methyl methacrylate in 1:2	Sustained release	
Kollicoat SR 30 D	Sustained release	
HPMC acetate succinate	Enteric coating	[35]
Cellulose acetate phthalate (CAP)	Enteric coating	
Eudragit L 30 D 55	Enteric coating	
Eudragit FS 30 D	Enteric coating	
Chitosan	Coating	[36]
EC, Shellac, Cellulose Acetate Trimellate	Film forming unit	[18]

**Table 6:** Opacifiers and colorants are used in FC [12].

Class	Examples
Natural colorants	Beta-carotene, riboflavin, carmine lake
Water soluble dyes	FD&C yellow no 5 lake, FD&C blue no 2 lakes
Inorganic Pigments	Titanium dioxide, iron oxides
D&C lakes	D&C red no 30 lake, D&C yellow no 10 lake
FD&C lakes	FD&C yellow no 5 lake. FD&C blue no 2 lakes

**Table 7:** Effect of plasticizer and pigments in FC [12].

Properties of the Films	Impact of Increased Concentration	
	Plasticizer	Pigment
Elastic modulus	Reduced	Increased
Tensile strength	Reduced	Reduced
Film Permeability	It depends on the physicochemical properties of the plasticizer used.	Decreased. The pigment volume reaches a critical concentration.
Hiding power	Little or no effect	Increased, but is dependent upon the refractive index and light absorption characters of the pigment
Viscous nature of coating material	Increases, but is directly related to plasticizer molecular weight.	Increased
Tg temperature	Reduced	Slight or no effect
Adhesion of the films	Generally, increases under ideal conditions	Slightly affected

**Table 8:** Some of the marketed products were summarized.

Type of Tablet	Example	Brand Name	Manufacturer
Enteric-coated tablet	Naproxen	Naprosyn	Roche Palo
SC tablet	Conjugated estrogen	Premarin	Wyeth Ltd.
FC tablet	Diclofenac	Voltaren	Novartis P’ceuticals [109]
SC	mebeverine hydrochloride	Colofac	Mylan
Enteric-coated	Misoprostol	Cytotec	Pfizer Medical Information—the US
Enteric-coated	Rabeprazole	Rabecid	Highnoon

## Data Availability

Not applicable.
